# In vitro and in vivo interactions of selected nanoparticles with rodent serum proteins and their consequences in biokinetics

**DOI:** 10.3762/bjnano.5.180

**Published:** 2014-10-02

**Authors:** Wolfgang G Kreyling, Stefanie Fertsch-Gapp, Martin Schäffler, Blair D Johnston, Nadine Haberl, Christian Pfeiffer, Jörg Diendorf, Carsten Schleh, Stephanie Hirn, Manuela Semmler-Behnke, Matthias Epple, Wolfgang J Parak

**Affiliations:** 1 Institute of Lung Biology and Disease, Helmholtz Center Munich, 85764 Neuherberg/Munich, Germany; 2 Institute of Epidemiology 2, Helmholtz Center Munich, 85764 Neuherberg/Munich, Germany; 3 Adolphe Merkle Institute, Université de Fribourg, 1723 Marly, Switzerland; 4 Walter Brendel Centre of Experimental Medicine, Ludwig-Maximilians-Universität München, 81377 Munich, Germany; 5 Fachbereich Physik, Philipps Universität Marburg, 35037 Marburg, Germany; 6 Inorganic Chemistry and Center of Nanointegration Duisburg-Essen (CeNIDE), University of Duisburg-Essen, 45117 Essen, Germany; 7 Berufsgenossenschaft Holz und Metall, 80809 München, Germany; 8 Bavarian Health and Food Safety Authority, 85762 Oberschleissheim, Germany

**Keywords:** biokinetics, gold nanoparticles, protein corona, protein–nanoparticle conjugate, serum protein binding, surface modification

## Abstract

When particles incorporated within a mammalian organism come into contact with body fluids they will bind to soluble proteins or those within cellular membranes forming what is called a protein corona. This binding process is very complex and highly dynamic due to the plethora of proteins with different affinities and fractions in different body fluids and the large variation of compounds and structures of the particle surface. Interestingly, in the case of nanoparticles (NP) this protein corona is well suited to provide a guiding vehicle of translocation within body fluids and across membranes. This NP translocation may subsequently lead to accumulation in various organs and tissues and their respective cell types that are not expected to accumulate such tiny foreign bodies. Because of this unprecedented NP accumulation, potentially adverse biological responses in tissues and cells cannot be neglected a priori but require thorough investigations. Therefore, we studied the interactions and protein binding kinetics of blood serum proteins with a number of engineered NP as a function of their physicochemical properties. Here we show by in vitro incubation tests that the binding capacity of different engineered NP (polystyrene, elemental carbon) for selected serum proteins depends strongly on the NP size and the properties of engineered surface modifications. In the following attempt, we studied systematically the effect of the size (5, 15, 80 nm) of gold spheres (AuNP), surface-modified with the same ionic ligand; as well as 5 nm AuNP with five different surface modifications on the binding to serum proteins by using proteomics analyses. We found that the binding of numerous serum proteins depended strongly on the physicochemical properties of the AuNP. These in vitro results helped us substantially in the interpretation of our numerous in vivo biokinetics studies performed in rodents using the same NP. These had shown that not only the physicochemical properties determined the AuNP translocation from the organ of intake towards blood circulation and subsequent accumulation in secondary organs and tissues but also the the transport across organ membranes depended on the route of AuNP application. Our in vitro protein binding studies support the notion that the observed differences in in vivo biokinetics are mediated by the NP protein corona and its dynamical change during AuNP translocation in fluids and across membranes within the organism.

## Introduction

Like any foreign material that enters into the organism, incorporated nanoparticles (NP) will bind with proteins of body fluids and receptor proteins of cellular and organ membranes. Binding of those proteins may cover the entire surface of the NP such that in the following these proteins are interacting with their biological vicinity and, hence, the subsequent fate of the NP is determined by the types of proteins covering the NP. In other words, the physicochemical properties of the NP play an essential role during protein binding but the biokinetics is largely determined by the covering proteins and their dynamic exchange in various biological fluids. This protein coating used to be called opsonisation in the past but now the popular name is “protein corona”. This protein corona is likely to be highly dynamic and to change in different body fluids. Furthermore, the most abundant proteins may bind first but get replaced by higher affinity proteins [1–3].

Hence, already more than a decade ago it has been hypothesized that soluble proteins in body fluids, such as blood or the epithelial lining fluid of the lungs, may not only bind to incorporated nanoparticles but such conjugates will serve as ferry boats navigated by interactions of those proteins with other proteins including those in membranes. Hence, these NP–protein conjugates behave like Trojan horses allowing nanoparticles to float in body fluids, cross cellular and/or tissue membranes, thereby reaching organs and tissues that were not expected to accumulate and retain these NP. Proteins bind also to submicrometer- or micrometer-sized particles, but the peculiar behavior of translocating protein–NP conjugates within body fluids and across organ membranes does not occur for these larger particles since their masses are too large for protein-mediated transport as we recently reviewed [4]. Although it was well known before that proteins bind to any incorporated particle, we expected that the small size of the NP similar to or slightly larger than the size of proteins will allow for protein-mediated transport. The latter would be unique for NP and would not be possible for larger particles in the size range of micrometers. Here, we have focused on the biokinetics of incorporated NP and did not consider any toxicological interactions between the NP and proteins and their reaction partners, which is currently a large scientific field studied by many researchers.

## Review

### Binding of serum proteins to NP and formation of protein–NP complexes

#### Binding of selected proteins

**Figure 1:**
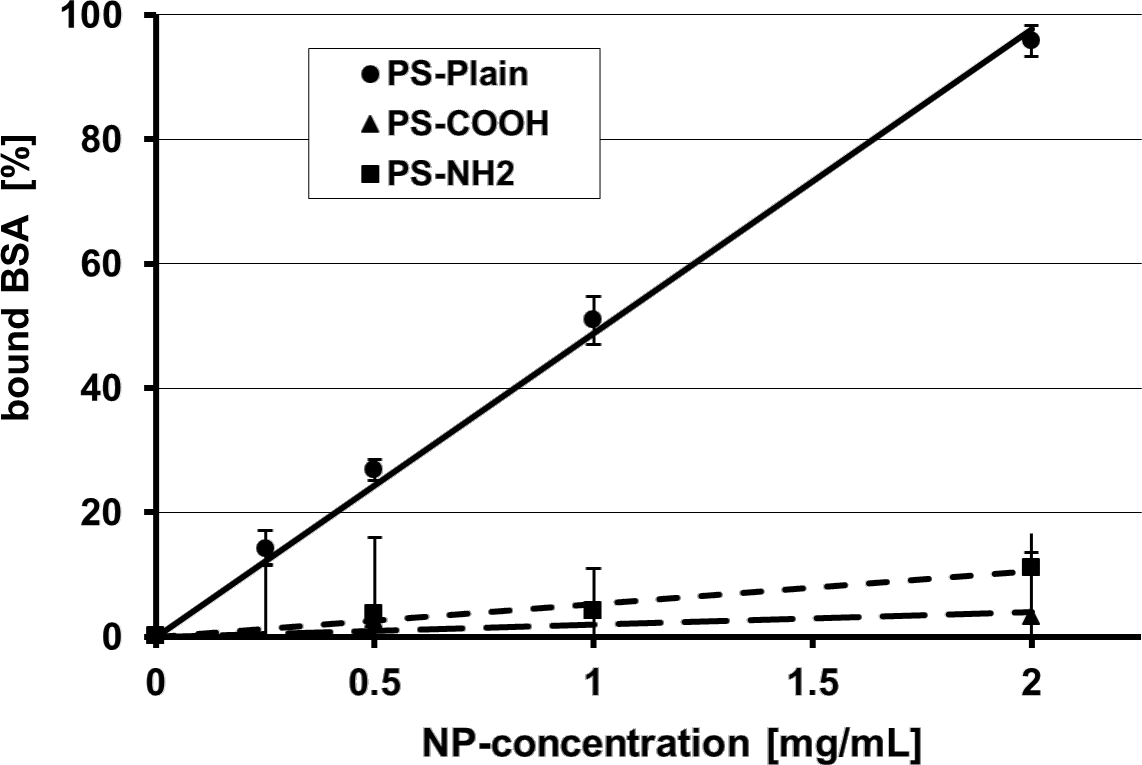
Bovine serum albumin (BSA) depletion in the supernatant after BSA separation from the 50 nm Polystyrene (PS) NP–protein complexes (PS-Plain: neutral charge, PS-COOH: negative charge by carboxyl groups, PS-NH_2_: positive charge by amino groups) depending on the NP dose; the error bars show the standard deviation). Each line represents the linear regression forced through the coordinate origin with the slope representing the binding index (BI). BSA binds with a much higher BI to hydrophobic, neutrally charged PS-Plain NP than the two other hydrophilic PS-NP. Reproduced with permission from [5]. Copyright 2011 Informa Plc.

First, we studied the parameters that determine the in vitro binding kinetics of selected NP during a one-hour incubation in simple phosphate-buffered saline (PBS) solutions in which a given protein was dissolved at 0.4 mg/mL; details are given in [5]. We chose nano-sized and submicrometer-sized carbon black versus 50 nm monodisperse polystyrene NP with surface modifications of either carboxyl groups (negative charge), or amino groups (positive charge) or plain surface (neutral charge) as measured by their zeta potential and agglomeration behavior [5]. The proteins chosen were albumin, transferrin and apolipoprotein A-1, which exist both in blood serum and in the lung epithelial lining fluid. Protein concentrations before and after NP incubation were determined by a depletion method using the Bio-Rad protein assay. In all cases, a linear correlation of the added amount of NP and the amount of bound proteins was found and was described quantitatively by binding indices (BI, Figure 1).

Hydrodynamic size distributions determined by dynamic light scattering (DLS) and zeta potentials were determined for all particles before and after incubation and showed varying agglomeration during incubation; in addition the protein coverage on the surface of each NP was estimated [5]. Table 1 summarizes the BI relative to the NP mass or NP surface area of all three PS-NP and the two carbon black Printex 90 and Printex G particles for all three proteins studied. The hydrophobic NP, PS-Plain and Printex 90 show much higher NP-mass based BI_mass_ for all three proteins than the hydrophilic PS-NP. Interestingly, the mass-based BI_mass_ of Printex G with all three proteins is also low because of the very small surface area of the incubated sub-micrometer sized particles. When calculating the BI relative to the surface areas of the incubated particles then the BI_surf_ values of all three hydrophobic particles are high but the BI_surf_ values of the hydrophilic NP remain low. We also estimated the surface coverage of the three proteins on the particle surface and found that in case of the hydrophobic particles the coverage was rather complete whereas only a very tiny surface percentage of the hydrophilic particles was covered (Table 1).

**Table 1:** Mass and surface area related to binding indices BI of the NP to the three different proteins, albumin, transferrin and apolipoprotein A-1. Reproduced with permission from [5]. Copyright 2011 Informa Plc. Diameter refers to the geometrical diameter as observed with transmission electron microscopy (TEM), and does not take into account the hydration shell and adsorbed counter ions [6].

			BI_mass_ (% protein/mg NP)			BI_surface_ (% protein/m^2^ NP)			surface coverage (%)		

	diameter (nm)	specific surface area (m^2^/g)	BSA	transferrin	Apo A-1	BSA	transferrin	Apo A-1	BSA	transferrin	Apo A-1

PS–Plain	50	120	49	56	43	407	466	359	66	≈100	269/76
PS–COOH	50	120	2	13	13	17	104	116	<1	4	81/23
PS–NH_2_	50	120	5	5	<0	44	39	<0	<1	12	0
Printex G	50	30	9	12	11	313	398	360	≈27	≈62	≈100
Printex 90	14	300	62	57	42	205	269	139	≈8	≈71	≈100

#### Serum protein binding during in vitro incubation of differently sized AuNP

In a second attempt we investigated the binding of the proteins after an in vitro 24-hour incubation of monodisperse, negatively charged gold spheres (AuNP) of 5, 15 and 80 nm core diameter and surface modified with bis(*p*-sulfonatophenyl)phenylphosphine in diluted mouse serum in PBS (1:50 v/v) by a two-step analysis: proteomic protein identification and quantitative protein biochemistry [7]. The mass concentrations of all AuNP varied from 6.7–200 µg/mL in steps of a factor of three, corresponding to the accordingly calculated AuNP surface area concentration (1–120 cm^2^/mL); thus, we obtained surface area concentrations between 10–40 cm^2^/mL of all three AuNP. Measurements by DLS before and after the AuNP incubation showed an increase of the hydrodynamic size distribution and a less negative zeta potential as a result of the formation of the protein corona. Additional TEM analyses proved that AuNP agglomeration was detectable but low, i.e., the changes in the hydrodynamic size distributions were mainly caused by the protein corona formation [7]. The proteins adsorbed to AuNP were separated from non-adsorbed proteins by a threefold centrifugation–washing process (each ultracentrifugation at 165,000*g* up to one hour for 5 nm and 15 nm AuNP; 80 nm AuNP were centrifuged and washed at 1000*g* for 20 min each as their mass assured a rapid precipitation at this centrifugal force). This was followed by protein detachment from the AuNP of the first pellet (by using a lysis buffer based on lithium dodecyl sulfate) and subsequently protein-mass-separation by gel electrophoresis. Negative controls were performed on the test tube used for the last washing step as well as on control incubations without any AuNP. For protein identification a MALDI-TOF MS proteomics analyzer was used. Furthermore, proteins in gel bands were analyzed quantitatively by protein densitometry. Only predominantly binding serum proteins could be analyzed by this quantitative approach.

**Figure 2:**
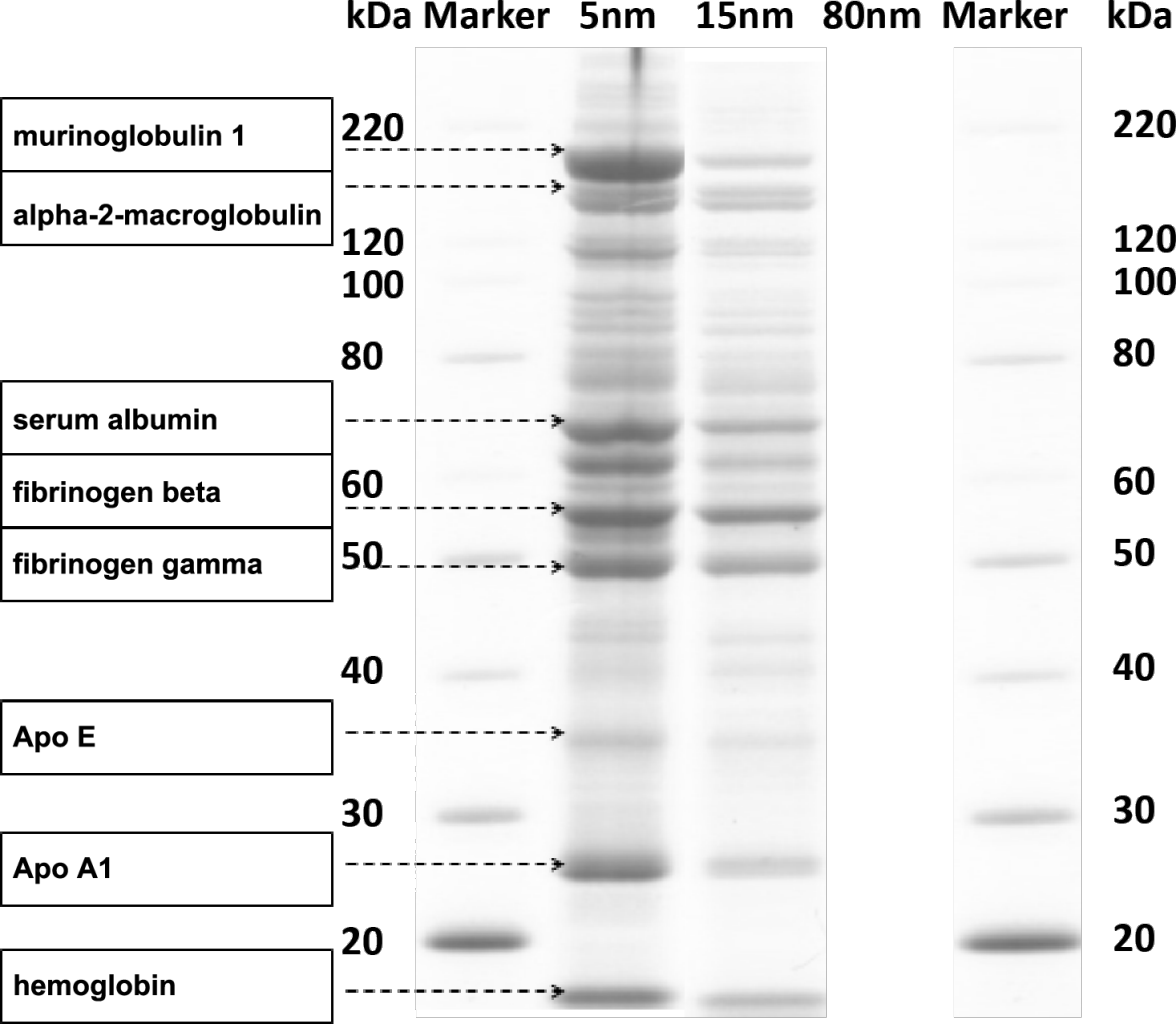
The size-selective separation of mouse serum proteins in the corona of the three differently sized AuNP are shown in the different gel lanes (after incubation and washing). Molecular weights (kDa) derived from the marker proteins are given at the left and right side. Labeled proteins were identified by MALDI–TOF MS. The number of bands and quantitative amount of adsorbed proteins differed between 5, 15 and 80 nm AuNP. Band intensities of adsorbed proteins decreased with increasing AuNP sizes. Reproduced with permission from [7]. Copyright 2013 IOP Publishing.

A typical electrophoresis gel visualized by Coomassie staining is given in Figure 2, which shows a variety of proteins present in the corona of the three differently sized AuNP. Proteins cover the whole range of molecular weights from 20–220 kDa. With increasing AuNP sizes, the gel shows decreasing band intensities at the same mouse serum concentration and same AuNP mass. The individual protein patterns show some similarities but they are not identical (see below). Similarly prepared gels of serum protein samples without AuNP proved to show no proteins (negative control data not shown).

**Figure 3:**
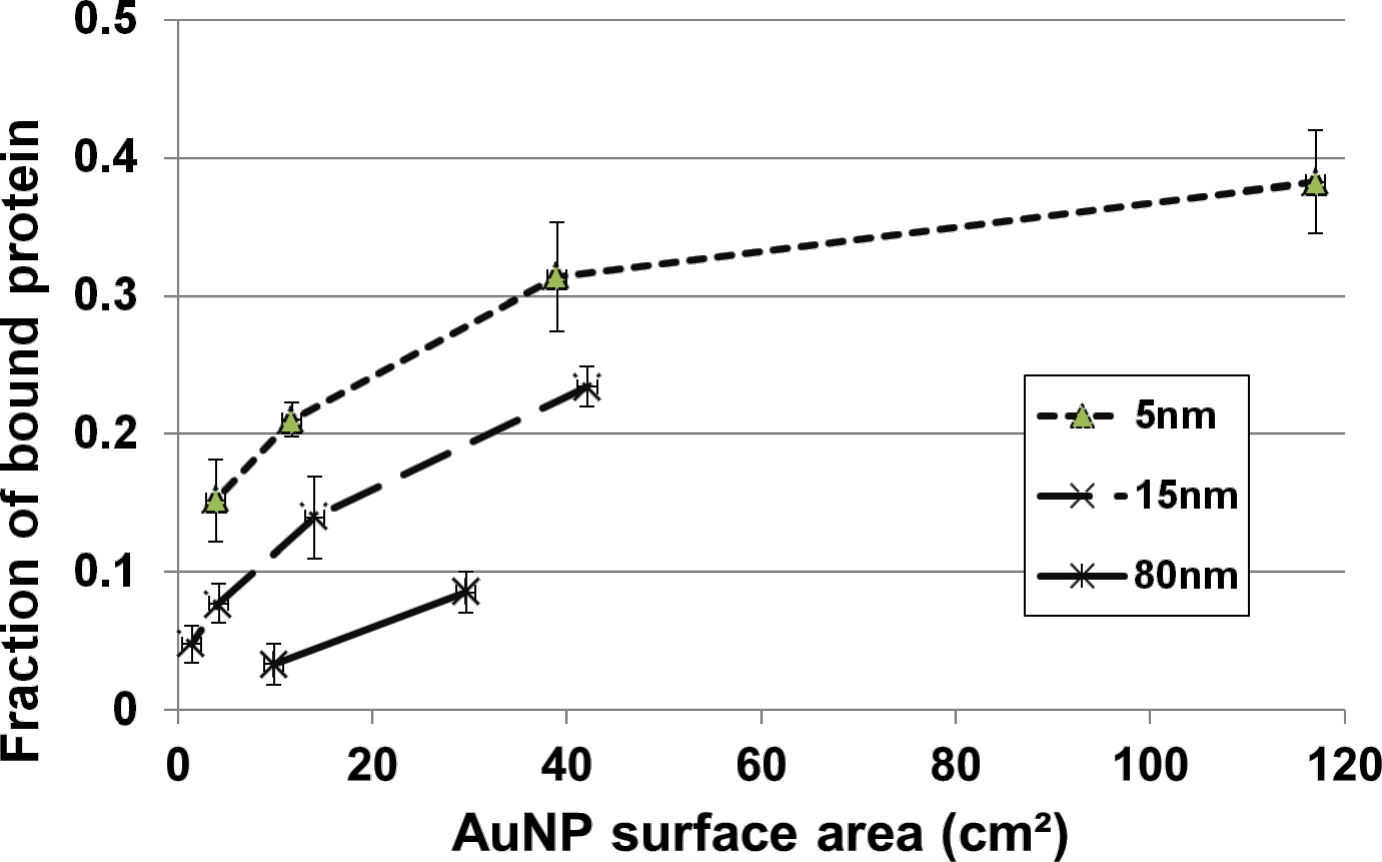
After 24 h incubation the fraction of totally available proteins binding to the AuNP is shown versus the calculated total surface area of all AuNP (as a sum of the individual surface areas of the single AuNP; mean ± SEM; *n* = 6). Reproduced with permission from [7]. Copyright 2013 IOP Publishing.

In a next step the role of the AuNP surface area available for protein adsorption was studied since the same mass of smaller AuNP have a larger surface area compared to large AuNP. Different concentrations of AuNP suspensions were used representing different available AuNP surface areas (Figure 3). Based on the total surface area of all AuNP expressed as the sum of the individual surface areas of all monodisperse AuNP, the three AuNP showed size-dependent protein-binding capacities for given total surface areas (Figure 3). For the same total surface area 5 nm AuNP bound a much higher total protein fraction compared to 15 nm AuNP and those bound a higher protein fraction than the 80 nm AuNP.

In other words, fewer proteins bind to the 80 nm AuNP surface area, which has the least surface curvature, compared to the smaller AuNP. This result is in agreement with the notion that the rather plain surface of the “big” 80 nm AuNP interacts only to a lesser extent with the three-dimensional structure of a protein and the orientation of its binding sites compared to a small AuNP (with a high curvature). These results indicate that the higher the surface curvature of the AuNP, the higher the protein binding capacity per surface area. Protein binding sites and their orientation and possible protein denaturation may contribute to this result.

Of the 82 different proteins that were detected once on the three AuNP we found 40 on the 5 nm AuNP, 35 on the 15 nm AuNP and 19 on the 80 nm AuNP (more details are available in the supplementary information of [7]). When considering only those proteins which were detected twice or more in order to improve the confidence of detection, 24 reproducibly detected proteins were identified on the three differently sized AuNP: 17 different proteins on the 5 nm AuNP, 17 on the 15 nm AuNP and 5 individual proteins on the 80 nm AuNP (Table 2). Proteins which were detected at least twice on each of all three differently sized AuNP were hemoglobin (oxygen transporter protein in the circulation), serum albumin (most abundant blood multi-functional protein), fibrinogen beta (modulation of blood coagulation and opsonisation of foreign bodies [8]), and apolipoprotein E (ApoE) (mediating protein for transcytosis across biological membranes [9–10]). More detected proteins were previously presented and discussed by Schäffler et al. [7]. In summary, multiple serum proteins were identified qualitatively by proteomic methodologies and quantitatively determined by protein biochemistry demonstrating that the composition of the protein corona in mouse serum depends on the size, surface area and curvature of the AuNP [7].

**Table 2:** Selection of murine serum proteins bound to the differently sized AuNP after 24 h of incubation. A total of nine SDS gels per AuNP were investigated by using proteome analysis (MALDI–TOF MS). Only proteins are listed that were detected at least twice.

	independent detections per protein				

protein (*n* = 9)	5 nm AuNP	15 nm AuNP	80 nm AuNP	moluclar weight (kDa)	function

serum albumin	9	8	3	69	osmotic pressure
hemoglobin	8	8	4	15	oxygen transport
fibrinogen beta	8	4	3	55	coagulation, immune response
Apo E	6	5	2	36	transport and cell uptake
Apo A1	9	7	—	30	metabolism
murinoglobulin 1	8	6	—	166	immune response
complement C3	7	8	—	186	immune response
alpha-2-macroglobulin	7	2	—	166	immune response
gelsolin	5	6	—	86	actin regulation (assembly)
fibrinogen gamma	5	4	—	50	coagulation, immune response
fibronectin	3	5	—	276	growth, migration and differentiation
Apo A4	6	—	—	45	hepatic transcellular lipid transport
Apo A2	5	—	—	11	transport
plasminogen	4	—	—	93	peptidase
myosin-4	2	—	—	224	muscle contraction
lg mu chain C region secreted form	2	—	—	50	immune response
alpha-1-antitrypsin	2	—	—	46	protease inhibitor
thrombospondin	—	3	—	134	immune response
complement b	—	2	—	86	immune response
plasma protease C1 inhibitor	—	2	—	56	protease
antitrombin III	—	4	—	52	protease inhibitor
actin	—	3	—	42	cell mobility
vitronectin	—	2	—	56	proteolysis regulation
protein spt 2 homologue	—	—	2	75	transcription

#### Serum protein binding of 5 nm sized AuNP with different surface modifications

**Figure 4:**
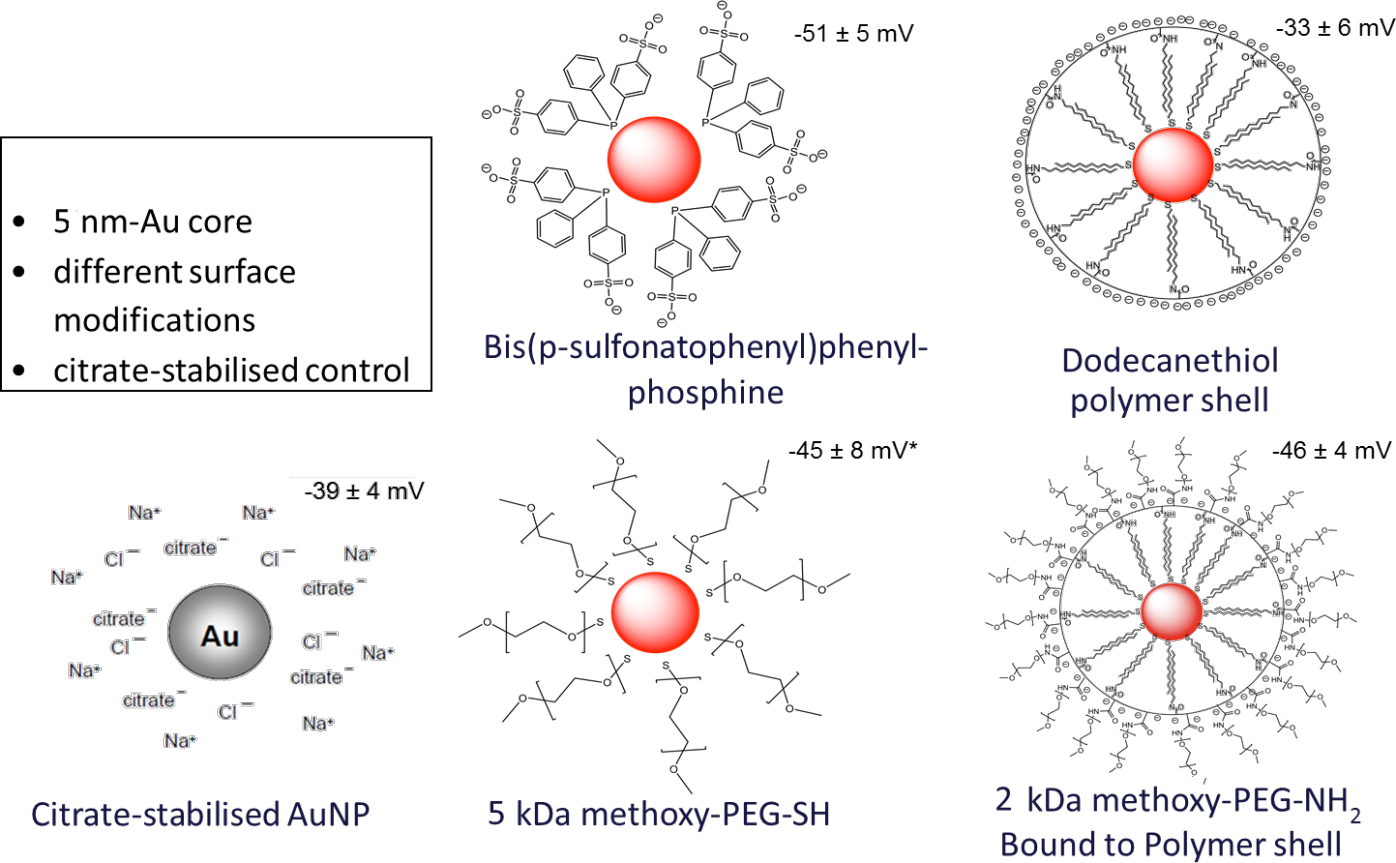
Spherical monodisperse AuNP of 5 nm core diameter with five different ligands. The zeta potential in millivolts is given for each NP.

In our next attempt we hypothesized that the configuration of the protein corona (composition, size, geometric orientation) depends on the surface ligands present on the AuNP. Therefore, we studied the role of five different surface modifications on monodisperse AuNP of 5 nm core diameter (i.e., the diameter as determined by transmission electron microscopy). As surface ligands we chose: citric acid (as supplied by the manufacturer), bis(*p*-sulfonatophenyl)phenylphosphine, a thiol-PEG, and an alkanethiol-ligand polymer shell with or without an additionally bound amino-PEG. The characterization of NPs with similar surface coatings has been reported in two previous papers [8–9]. Schematic sketches of the AuNP with their surface modifications are shown in Figure 4.

Besides the previous physicochemical characterizations [8], all AuNP used in these experiments were thoroughly characterized through TEM (size distribution), UV–vis spectroscopy (absorbance), dynamic light scattering (DLS, hydrodynamic diameter), differential centrifugal sedimentation (DCS) and atomic force microscopy (AFM, morphology, height and agglomeration). As a result, after incubation in either diluted mouse serum in PBS (1:50 (v/v) or four-times concentrated murine broncho-alveolar lavage (BAL) fluid based on Mg and Ca ion free PBS (see below) the size distributions of the hydrodynamic diameters of all five AuNP were compared to the corresponding AuNP suspensions in either PBS or distilled water. As an example, the DCS measurements showed that the spectral size shifts, from 5 nm AuNP in PBS or distilled water to those after incubation in diluted mouse serum or BAL fluid, did only increase by less than a factor of 2 of the median diameter prior to incubation. This contrasts DLS and AFM measurements, which yielded larger diameters of the AuNP surface-modified with large molecules (bis(*p*-sulfonatophenyl)phenylphosphine, thiol-PEG, polymer shell with or without amino-PEG) even in PBS or distilled water. The latter demonstrates that AFM and DLS include the surface molecules into measurement of the size, while DCS does not (Johnston et al. in preparation).

**Figure 5:**
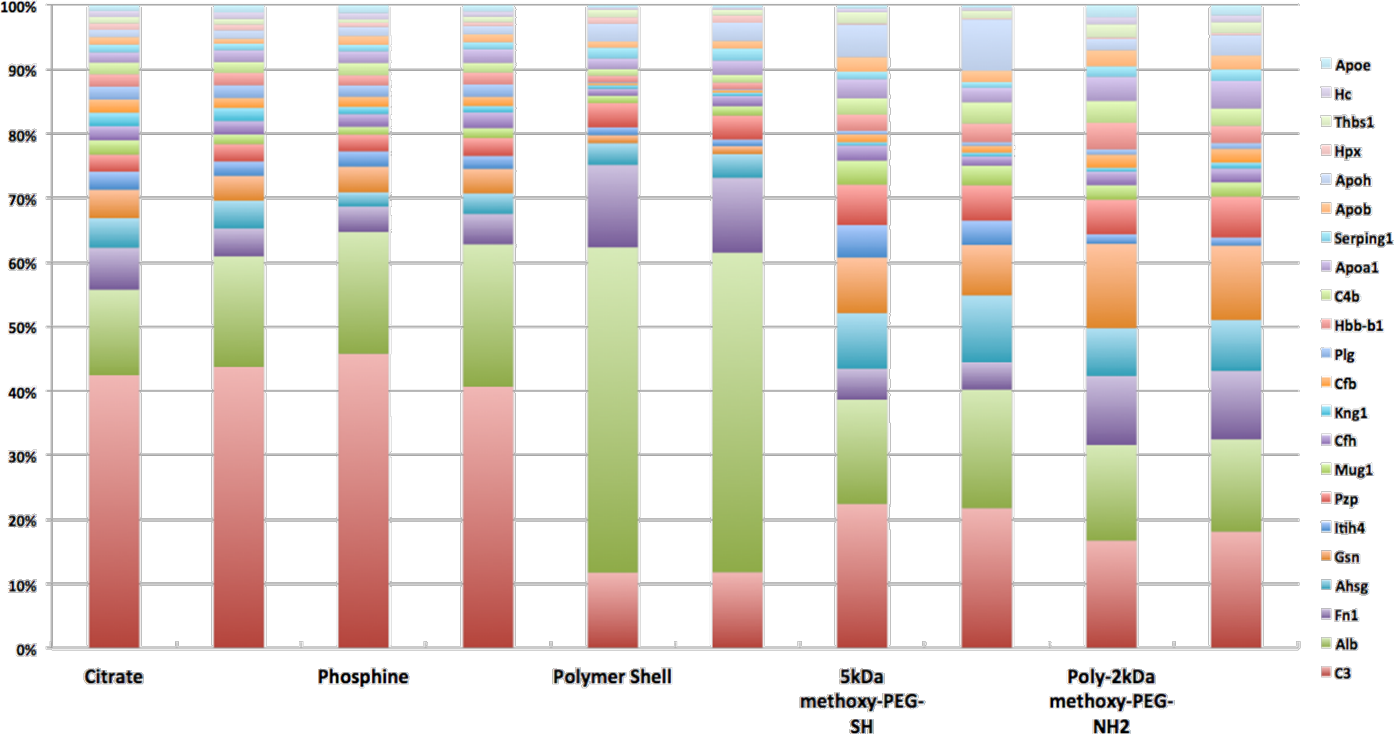
Protein percentages in cumulative stacks of the most abundant proteins bound to either of all five AuNP with different surface modifications in mouse serum after 15 min incubation. The order of the proteins from the bottom to the top of the stack were chosen by the protein with the highest binding percentage to the control AuNP (citrate stabilized) followed by proteins of successively lower binding percentages from bottom to top. Protein abbreviations on the right column from top to bottom: Apoe: apolipoprotein E; Hc: hemolytic complement; Thbs1: thrombospondin 1; HPX: hemopexin; Apoh: apolipoprotein H; Apob: apolipoprotein B; Serping1: serine (or cysteine) peptidase inhibitor; Apoa1: apolipoprotein A1; C4b: complement component 4b; Hbb-b1: hemoglobin, beta adult major chain; Plg: plasminogen; Cfb: complement factor b; Kng1: kininogen 1; Cfh: complement component factor h; Mug1: murinoglobulin 1; Pzp: pregnancy zone protein; Itih4: inter alpha-trypsin inhibitor, heavy chain 4; Gsn: gelsolin; Ahsg: alpha-2-HS-glycoprotein; Fn1: fibronectin 1; Alb: albumin; C3: complement component 3.

AuNP were incubated for 15 min or 24 h in either diluted mouse serum or murine broncho-alveolar lavage fluid. After incubation the suspension was centrifuged (75000*g* for 120 min) and washed three times to remove non-bound proteins. Subsequently, one fraction was used for the determination of the hydrodynamic diameter distribution after incubation and from the other fraction proteins were separated from the AuNP by a sodium dodecyl sulfate (SDS)-lysis buffer and SDS-polyacrylyamide gel electrophoresis (PAGE) was carried out. Thereafter, gel lanes were dissected in smaller units and prepared for liquid chromatography mass spectroscopy/mass spectroscopy (LC–MS/MS analysis). Figure 5 shows the protein patterns of the most abundant proteins for all five AuNP in mouse serum after 15 min of incubation. Figure 5 shows the very good reproducibility of two independent LC–MS/MS measurements for each of all five AuNP.

### Biokinetics of AuNP after administration via three routes of intake

The above mentioned in vitro studies were designed to provide a better understanding about the role of proteins in blood and other body fluids on the biokinetics of NP in the organism after various routes of intake. The in vivo studies represent the main focus of our research since the late 1990s. The knowledge about the biokinetics of biopersistent NP is required when assessing their toxicity in various organs and tissues and developing an understanding of their potential risks. When particles are taken up into the organism, they do not necessarily remain at their initial sites of intake. Instead they can undergo numerous transport processes within the various tissues of the organ of intake and secondary organs and tissues.

**Figure 6:**
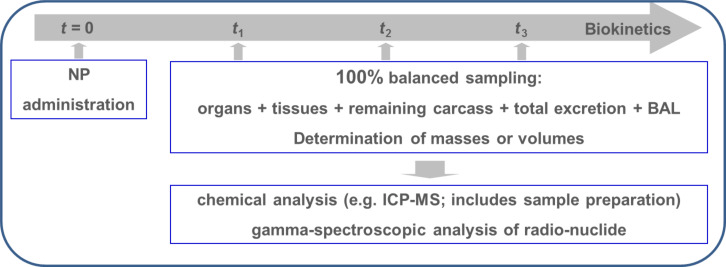
Schematics of the analysis of quantitative NP biokinetics. After particle administration at time *t* = 0 the whole urinary and fecal excretions are collected separately. Animals are euthanized at times *t*_1_, *t*_2_, *t*_3_, etc., and organs and tissues of interest and the entire remaining carcass are sampled (100% balanced sampling) and their weights or volumes are determined. Chemical analysis requires additional, separate steps of sample preparation. When particles are radio-labeled, total organs and tissue samples including the entire remaining carcass will be analyzed directly by using gamma spectrometry without any further preparation. Adapted from [4].

Therefore, we developed and applied an in vivo assay on the quantitative biokinetics of biopersistent NP in the organism of experimental rodents. It aims for an overall quantitative estimate of the total amount of the administered particles biodistributed within and out of the body. The analysis of quantitative biokinetics can be performed after the NP administration by any route of intake, via the respiratory tract, the gastrointestinal tract, the blood circulation or the skin. Basically, the total amount of particles administered and retained in the entire body is estimated at different time points after exposure and, in addition, it includes the analysis of total excretion, Figure 6. Therefore, the total of all NP incorporated are included in this analysis: not only the NP distributions in organs and tissues of interest, but also NP in the remaining carcass and those in the excretion. As a result, the administered NP are 100% balanced of and a complete and detailed quantitative analysis of their biokinetics is obtained providing data on the fraction of NP per organ or tissue but also NP mass- or NP surface- or number concentrations per gram of organ or tissue (or those of other metrics) .

We applied this in vivo assay and investigated systematically the biokinetics of a suite of monodisperse, spherical gold NP (AuNP) with Au core sizes of 1.4, 2.8, 5, 18, 80 and 200 nm in healthy female adult Wistar Kyoto rats after administration via three routes: intratracheal instillation into the lungs, intravenous injection into blood and intra-esophageal instillation into the gastro-intestinal tract. The AuNP were synthesized and surface-modified not only with small ionic ligands such as sulfonated triphenylphosphine, amino groups, or carboxyl groups, but also with polyethylene glycol (PEG) or other polymers or polyelectrolytes and, finally, with tightly grafted plasma proteins (albumin or apolipoprotein E). All AuNP had been radioactively labeled with ^198^Au by neutron activation in a nuclear research reactor. Most of these studies were published in the following series of reports:

- biokinetics of ^198^AuNP of different small ionic ligand surface modification: after intratracheal instillation or intravenous injection of 1.4 nm and 18 nm ^198^AuNP; Semmler-Behnke et al. [10]; after intravenous injection of 1.4, 2.8, 5, 18, 80 and 200 nm ^198^AuNP; Hirn et al., [11]; after intra-esophageal instillation of 1.4, 2.8, 5, 18, 80 and 200 nm ^198^AuNP; Schleh et al. [12]; after intratracheal instillation of 1.4, 2.8, 5, 18, 80 and 200 nm ^198^AuNP Kreyling et al. [13],
- biokinetics of ^198^AuNP with PEG versus small ionic ligand surface modification are compared after intratracheal instillation or intravenous injection of 5 nm ^198^AuNP; Lipka et al. [14],
- biokinetics of protein–AuNP conjugates with albumin or apolipoprotein E versus polyelectrolytes and a small ionic ligand are compared after intravenous injection of 15 nm and 80 nm ^198^AuNP; Schäffler et al. [15],
- biokinetics of AuNP by double radio-labelling of Au cores (^198^Au) and their polymer surface coating (^111^In) with 4 nm core size; methodology was established: Ali et al. [16]; biokinetics after intravenous injection (Kreyling et al., manuscript submitted)

In this series of in vivo investigations we found similarities as well as significant differences between the various AuNP and their different physicochemical properties. Common to all AuNP administered to the lungs by intratracheal instillation was the predominant retention in the peripheral lungs [10]. In particular, more than 99% of AuNP with core diameters greater or equal than 18 nm were retained in the lungs after 24 h [13]. However, the smaller the AuNP were, the more they translocated into blood and accumulated in secondary organs such that about 5% of the 1.4 nm AuNP were translocated from the lungs into blood. This clearly indicates that the lungs are capable to trap and retain NP at least at those low doses of 1–10 µg per rodent lungs, even when the NP had been administered in a bolus of suspended AuNP via intratracheal instillation. Interestingly, translocation from lungs to blood increased proportionally with the inverse of the Au core diameter (i.e., the specific surface area (SSA) of these spherical AuNP) from 0.1% of 80 nm AuNP to 8% of 1.4 nm AuNP [13]. However, this SSA-dependency did not apply for the 200 nm AuNP. Furthermore, when considering how these translocated AuNP accumulated in different organs and tissues the fractions were similar for all AuNP and became independent of the AuNP size. Liver accumulated about 10% of these translocated AuNP, which was more than in other organs. However, the largest fraction of about 50% was accumulated in the remaining carcass consisting of the skeleton, soft tissue and skin. Estimating the skeletal AuNP fraction about 15% of the translocated AuNP were accumulated from blood to the bone marrow with its sensitive stem cell population. For more details see [10,13].

After the intravenous injection of AuNP suspensions a retention is expected in the organs of the mononuclear phagocyte system (MPS) with the largest fraction found in the liver. Indeed, more than 98% of AuNP with core diameters ≥ 18 nm were retained in the liver, particularly in Kupffer cells, and about 1–2% were retained in the spleen [11]. However, the smaller the AuNP were, the more they accumulated in other organs such that only 50% of 1.4 nm AuNP were retained in the liver. This decline was proportional to the inverse AuNP core diameter. This is in contrast to the SSA-independent liver accumulation for AuNP which had crossed from the lungs to blood after intratracheal instillation, as mentioned above. Furthermore and in contrast to the SSA-independent accumulation for AuNP which had crossed from the lungs to blood the accumulation in the remaining carcass declined drastically from 25% for 1.4 nm AuNP to less than 0.2% for 80 nm and 200 nm AuNP. Hence, when considering the inverse diameter (i.e., the SSA) the accumulation of the remainder increases sharply with SSA, which is a clearly different behavior compared to intratracheal instillation. For more details see [11].

After oral administration more than 99% of all AuNP were excreted via feces. Yet, absorption and translocation across the gut wall was clearly size dependent such that 0.4% of the negatively charged 1.4 nm and 2.8 nm AuNP crossed the gut wall while this fraction was smaller than 0.1% for all other AuNP. Most of these absorbed AuNP accumulated in the remaining carcass consisting of the skeleton, soft tissue and skin, which is consistent with the findings after intratracheal instillation. For more details see [12].

Twenty-four hours after administration via all three routes significant amount AuNP still circulated in blood with the actual fractions depending on the particle size. Highest fractions were always found for the smallest AuNP. These findings support the notion that the initially administered AuNP had bound blood proteins including those which prevented recognition of the phagocytes of the organs of the MPS. In this context we also studied the role of poly(ethyleneglycol) (PEG) surface modifications on the biokinetics of AuNP of 5 nm Au core size over 24 h [14]. Indeed we confirmed that applying long-chained PEG (10 kDa) prolonged the circulation time of 95% of the administered AuNP considerably after one hour, while the circulation time was prolonged for only 2% of sulfonated triphenylphosphine-modified AuNP of the same size. Yet, short-chained PEG (750 Da) did not yield such a prolongation indicating less stability of the PEG surface modification. Even for the long-chained PEG only 15% of the initially administered AuNP were still circulating after 24 h suggesting a gradual degradation of the PEG shell.

Our in vitro studies clearly demonstrated the rapid binding of serum proteins to AuNP. This is likely reflected in the in vivo biokinetics results found after intravenous injection which led to a predominant accumulation in the liver and Kupffer cell endocytosis. Yet, the fact that even after 24 h a significant fraction of AuNP still circulated in blood, points to a fraction of AuNP bound to proteins, which had not been recognized by the phagocytic cells of the MPS. The fact, that AuNP showed a completely different pattern of accumulation in the various organs and tissues after translocation across either the air–blood barrier of the lungs or the gut epithelium strongly suggests that those AuNP had bound to different proteins compared to those that bound to blood proteins after intravenous injection. It is plausible to assume that this dynamic protein exchange occurred during the translocation across membrane barriers by either transcellular endo- and exocytosis, or by paracellular transport mechanisms. While there are some in vitro studies suggesting protein exchange on NP surfaces during membrane crossing we are not aware of any in vivo studies having shown such exchange [17–19]. Hence, our in vivo studies together with our in vitro studies as well as other in vitro studies support the notion that protein binding on NP surfaces does undergo dynamic protein exchange when NP cross organ membranes.

In order to identify the role of individual proteins on the biokinetic fate of NP–protein conjugates we engineered such conjugates by using either albumin or apolipoprotein E grafted through polyelectrolyte links onto the surface of 15 nm or 80 nm AuNP prior to intravenous injection into mice [15]. Indeed, we observed remarkable differences in the biokinetics pattern during the first 48 h after administration. AuNP retention in the liver decreased drastically while AuNP retention in lungs and spleen jumped up ten-fold for both protein–AuNP conjugates of 15 nm AuNP core size when compared to citrate-stabilized AuNP of the same size. In case of albumin–AuNP conjugates, retention in the brain increased even 100-fold while the retention of apolipoprotein-E–AuNP conjugates in the brain was still 10-fold when compared to citrate-stabilized AuNP of the same size. Similarly, retention of both protein–AuNP conjugates was also increased 10-fold in the remaining carcass consisting of skeleton, soft tissues and skin. Interestingly, only small increases were seen for the conjugates with 80 nm AuNP core size indicating the relevance of the AuNP surface curvature and the binding orientation of the conjugated proteins. These results emphasize the determining role of distinct proteins on the biokinetics of grafted AuNP conjugates. However, these results also suggest that more research is required to better understand the mechanisms of how these proteins and others mediate the passage across organ membranes.

#### Precision-cut lung slices after intratracheal instillation of PVP-coated silver NP

Several studies already described adverse effects of silver nanoparticles (AgNP) on the lungs [20–25]. However, information about the effects of AgNP on diseased lungs is lacking. So, the aim of the current study was to investigate the effects of AgNP on lungs, which were subsequently incubated with lipopolysaccharide (LPS) as a simple model for a bacterial infection. In vivo pre-exposure of NP to the lungs leads to rapid clearance of the NP out of the conducting airways and the dissolution/dissociation of NP, but also to formation of the protein corona and numerous cellular interactions between the NP and the lung epithelium including functional and structural alterations of proteins by Ag ions and also AgNP translocation into interstitial spaces as described above. These interactions with proteins and cells and the changes in the lung caused by both AgNP and Ag ions are likely to modulate subsequent bacterial infections of the lungs in a way that can only be determined by an experimental design like the one described below. For this purpose, thin slices from rat lungs, so-called precision-cut lung slices (PCLS), were employed. In PCLS the lung structure with all proteins and important cell types is retained and the number of test animals can be reduced by creating multiple PCLS from one single lung. Thus, PCLS may represent a good ex vivo intermediate between in vitro and in vivo test systems. In the following we describe a pilot study anticipated to be followed by in-depth proteomics and cellular analyses of lung tissue exposed to AgNP and Ag ions as well as more realistic bacterial post-exposures.

We used monodisperse, spherical, PVP-coated AgNP with a size of 70 nm which had been carefully characterized [26–27]. The AgNP were dispersed in degassed distilled water and the hydrodynamic diameter distribution was verified by dynamic light scattering (DLS) measurements prior to intratracheal instillation in groups of four healthy, adult, female Wistar-Kyoto rats (8–10 weeks aged, body weight 180–200 g). Doses of freshly prepared AgNP were 50 µg and 250 µg per rat in 80 µL suspension. In addition, a dose of 250 µg AgNP in a six-month old AgNP suspension and the corresponding AgNP-free supernatant previously separated from AgNP by centrifugation (186000*g*, 30 min followed by ultrafiltration through a 15 kDa Amicon filter at 1500*g* for 15 min) that contained only the released Ag^+^ ions was also tested. PBS served as a negative control and 250 µg Ag^+^ ions from silver acetate served as a positive control.

Twenty-four hours after instillation, rats were killed under deep anaesthesia. The lungs were inflated at tidal volume with low-melting agarose, excised and fixed in ice-cold saline. Cylinders (8 mm diameter) of lung tissue were punched out and cut into 200 µm thick slices (precision-cut lung slices, PCLS). Four PCLS were incubated in full medium in each well of 24-well tissue culture plates. The plates were put into a 37 °C tissue culture incubator, and the melting agarose as well as tissue debris were removed by three washings of the medium within the next hour. In order to simulate a bacterial infection in a first attempt, LPS was added at increasing concentrations 1, 100, 500, 1000 and 5000 ng/mL into two wells for each concentration. Either after four or after twenty-four hours the viability of the PCLS was tested by lactate dehydrogenase (LDH) analysis for cell membrane damage and a WST-1 assay for mitochondrial activity in the cell culture supernatant as well as the release of the pro-inflammatory cytokines tumor necrosis factor alpha (TNF-α) and interleukin (IL-8) was analyzed.

After four hours, both viability tests demonstrated that the integral viability signals of the PCLS did not decrease with increasing LPS concentrations up to 5 µg/mL. Yet, after the second and third day, the viability decreased significantly (data not shown). Similarly, the protein content of the PCLS did not change for the same range of LPS concentrations during the first 24 hours (data not shown). Further discussion including cytokine release will be discussed below.

These ex vivo studies are complemented by in vivo studies, after instillation of the same AgNP at the same doses, on toxicological responses of rat lungs determined in broncho-alveolar lavage fluids by using the same endpoints as in the ex vivo studies described above [22]. Remarkably, the rather consistent findings of increased pro-inflammatory response in an AgNP dose-dependent manner as determined by several toxicological endpoints in the in vivo studies were much less pronounced and also inconsistently observed in the ex vivo PLCS studies. A possible explanation of the inconsistent results is the fact that the intratracheal instillation of the AgNP suspension led to a rather inhomogeneous deposition in the lungs of the rats. Furthermore, the PCLS represented a volume of lung tissue that was insufficient and did not reflect the overall AgNP distribution in the lungs and their response. This leads to a large variability in the observed measurements. In contrast, the broncho-alveolar lavages rinse most of the entire epithelial surface and, hence, BAL fluid collects most of the pro-inflammatory markers, which led to the rather consistent response depending on the AgNP dose.

**Figure 7:**
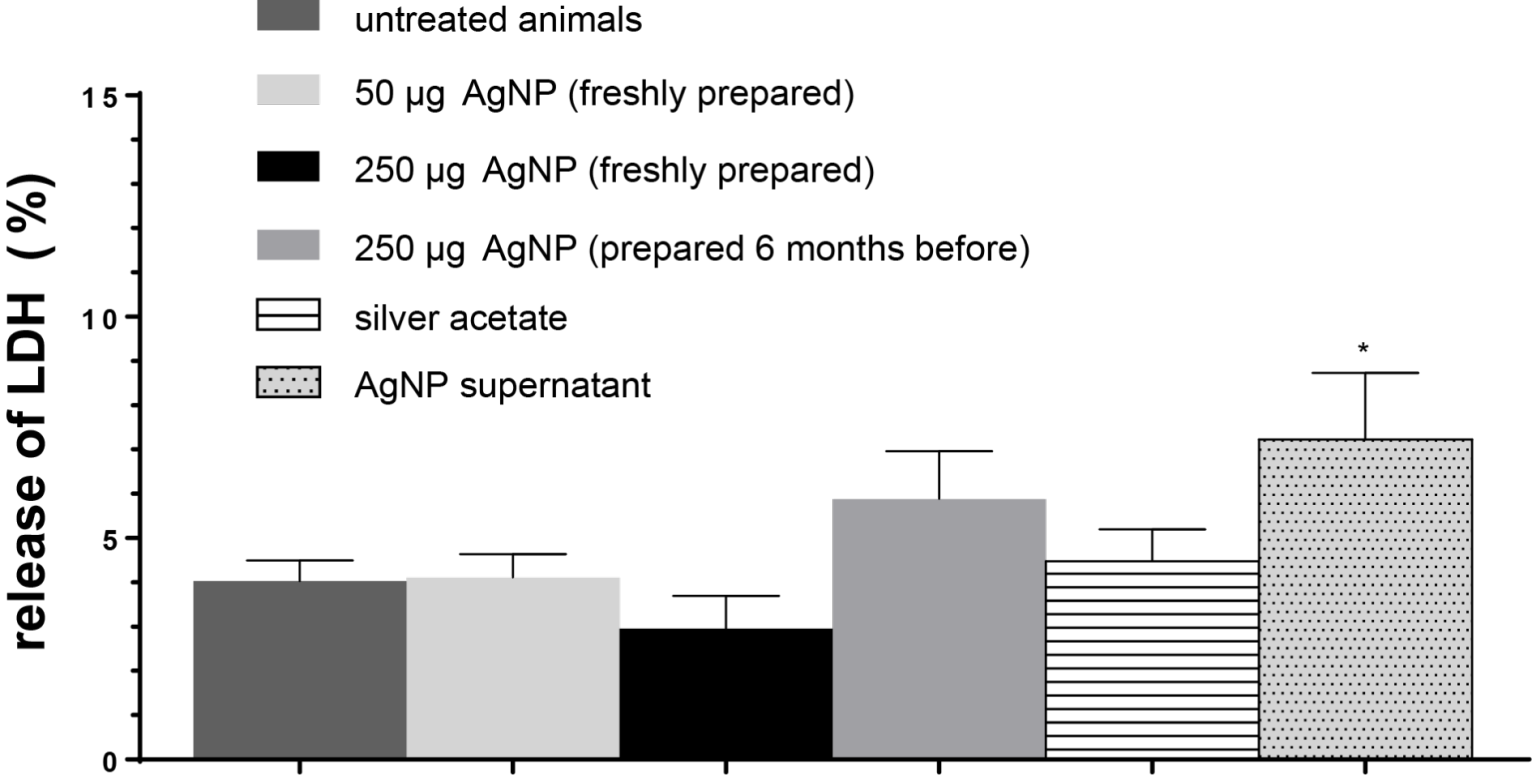
Release of LDH in the supernatant after incubation of PCLS in tissue culture medium for 4 h. The animals had been treated 24 h before either with 50 or 250 µg AgNP (freshly prepared or six months old) or silver acetate (250 µg Ag^+^) or AgNP-free supernatant after centrifugation of AgNP. The percentage of total LDH-release from PCLS lysed with Triton X-100 is displayed. Data are represented as mean values ± standard errors of the mean (SEM), with a sample size of *n* = 4.

In the following, we present results shedding light on the differences between the viability and the release of pro-inflammatory cytokines of PCLS exposed to either AgNP or dissolved Ag or mixtures of both. The LDH release of PCLS increased significantly when the rats were instilled with the aqueous supernatants of AgNP centrifugation compared to PCLS of untreated control rats (Figure 7). Consistently, LDH was also increased in PCLS from rats treated with six-month old, 250 µg AgNP. But this increase was not statistically significant.

This indicates that dissolved/dissociated Ag species which had been released from AgNP damaged the cell membrane of PCLS more than the Ag^+^ ions from silver acetate. However, mitochondrial changes did not change in between any of the groups of rats.

**Figure 8:**
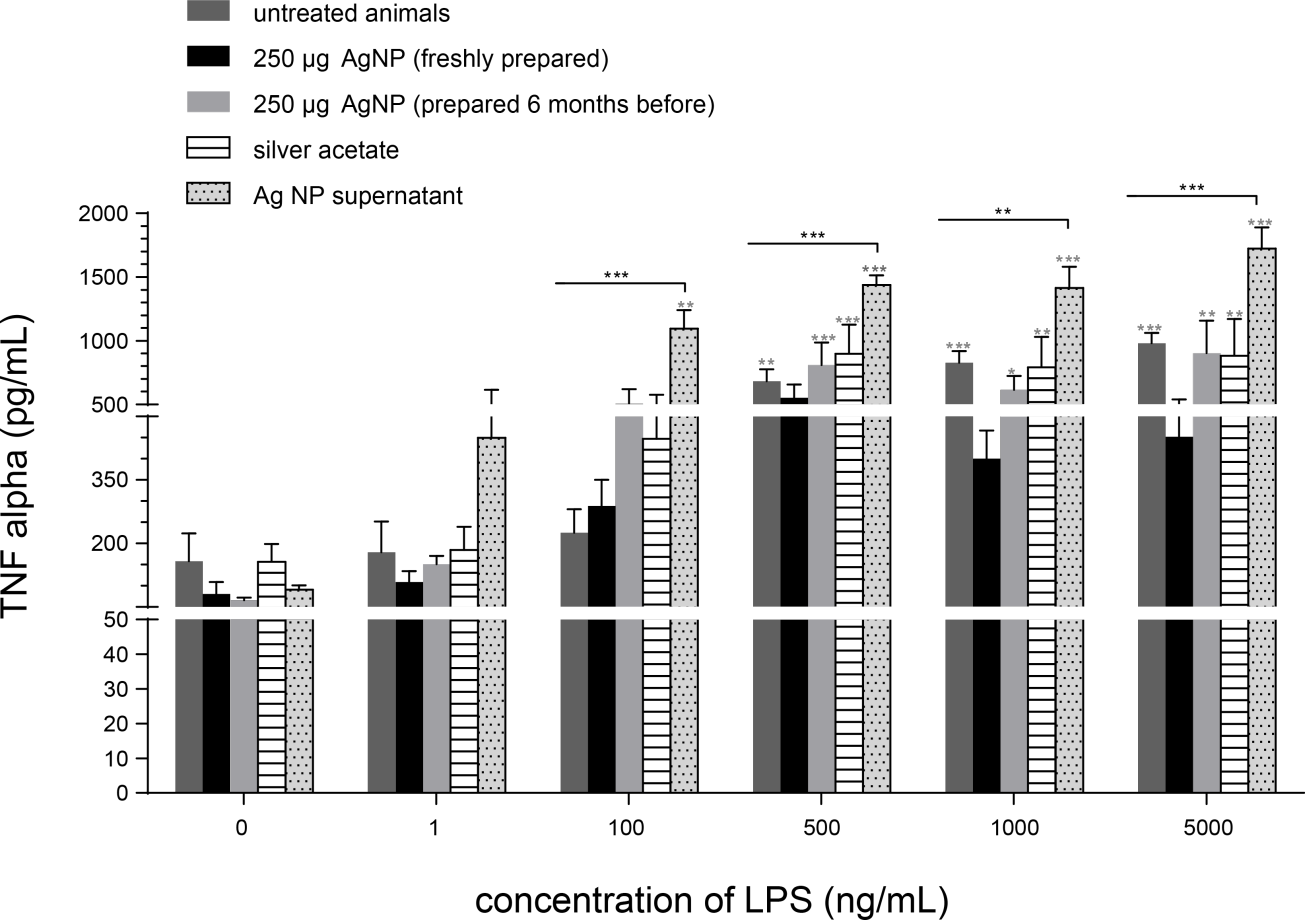
Release of TNF-α in the supernatant after 4 h of incubation of PCLS (of untreated animals or animals treated with 250 µg AgNP (freshly prepared or aged for six months), or silver acetate (250 µg Ag^+^) or AgNP-free supernatant) with increasing LPS-concentrations in the tissue culture medium. The instilled volume was always 80 µL. Data are represented as mean ± SEM; *n* = 4–7; *,**,***: significantly different from PCLS exposed to 0 ng/mL LPS; *,**,***: significantly different from PCLS of untreated animals; *p* < 0.05 (*), *p* < 0.01 (**), *p* < 0.001 (***).

Interestingly, the inflammatory response determined by TNF-α and IL-8 release increased significantly depending on the LPS dose in PCLS of rats that were exposed to suspensions containing 250 µg AgNP (Figure 8). It is quite striking that the TNF-α release of PCLS exposed to 250 µg AgNP prepared six months earlier was higher for each LPS concentration when compared to 250 µg of freshly prepared AgNP. Furthermore, for all LPS concentrations the TNF-α release of PCLS treated with 250 µg Ag in silver acetate solution was rather similar to those of the group treated with six-month old 250 µg AgNP. In addition, the highest TNF-α release of PCLS for all LPS concentrations was observed in PCLS that had been treated with the AgNP-free supernatant (after AgNP removal by centrifugation and filtration). This result indicates that in a six-month old AgNP suspension more dissolved/dissociated Ag species are present in comparison to a freshly prepared AgNP suspension [26]. In addition, these results are in line with the decreased viability of PCLS in Figure 8, which were treated with 250 µg AgNP in a six months old suspension or with AgNP free supernatant. Obviously, the dissolved/dissociated Ag species released from the AgNP in distilled water are more cytotoxic and more damaging to cell membranes than the Ag^+^ ions in the silver acetate solution.

**Figure 9:**
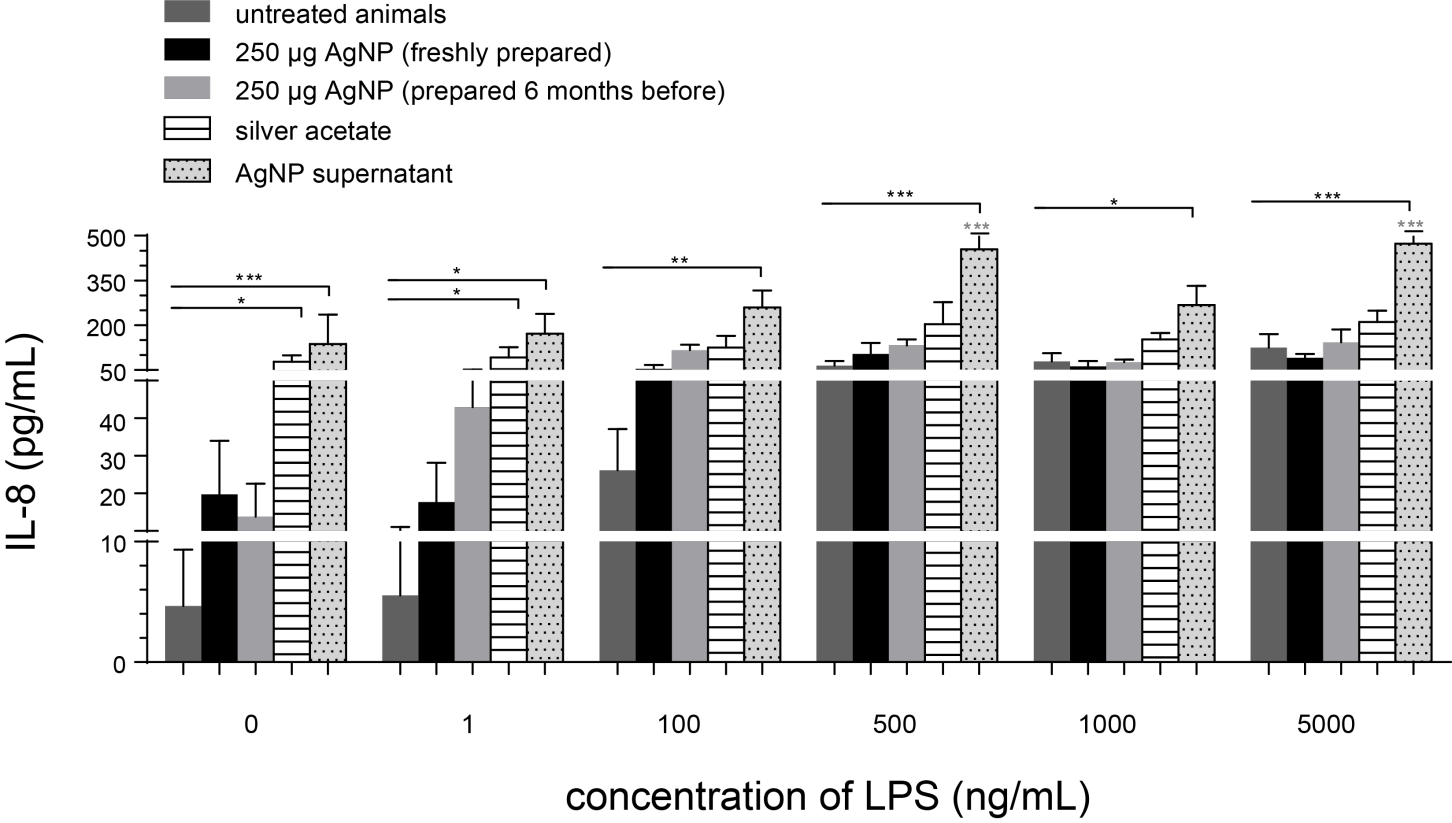
Release of IL-8 in the supernatant after 4 h of incubation of PCLS (from untreated animals or animals treated with 250 µg AgNP (freshly prepared or aged for six months), or silver acetate (250 µg Ag^+^) or AgNP-free supernatant) with increasing LPS-concentrations in the tissue culture medium. The instilled volume always was 80 µL. Data are represented as mean ± SEM; *n* = 4–7; *,**,***: significantly different from PCLS exposed to 0 ng/mL LPS; *,**,***: significantly different from PCLS of untreated animals; *p* < 0.05 (*), *p* < 0.01 (**), *p* < 0.001 (***).

A similar and, hence, consistent response pattern was observed when analyzing the IL-8 cytokine response of the PCLS of the various groups in Figure 9. Again, the highest increase of IL-8 was observed when rats had been treated with AgNP-free supernatant (after AgNP centrifugation) for all LPS concentrations applied.

During the six months of aging, about half of the AgNP mass becomes dissolved as was shown by Kittler and coworkers [26]. Hence, both the six-month old 250 µg AgNP suspension and the AgNP-free supernatant contained similar concentrations of Ag^+^ ions and/or dissociated Ag. Both, the AgNP suspension and the supernatant were instilled directly into the rat lungs. Upon contact with the epithelial lung lining fluid containing 0.9% NaCl the instillate was spread out and diluted such that Ag^+^ ions formed AgCl, which precipitated and possibly formed nano-sized AgCl particles [27]. These AgCl nanoparticles also underwent endocytosis in the lung epithelium. Subsequently they were dissolved/digested intracellularly probably faster than the engulfed AgNP that formed Ag^+^ ions or other reactive Ag species, which apparently caused the increased LDH release and the release of both cytokines [9,27]. Interestingly, instilled silver acetate showed a similar trend as the suspension of the six-month old AgNP, albeit not such a clearly increased release of LDH and cytokines. Note that silver acetate is fully dissociated in water and the Ag^+^ ions will form AgCl nanoparticles upon contact with the epithelial lung lining fluid as do those in the AgNP suspension or in the AgNP-free supernatant. This discrepancy suggests that the Ag dissociation during aging of the AgNP suspension is more complex than just forming Ag^+^ ions.

## Conclusion

Upon contact with body fluids such as blood serum NP bind soluble proteins according to their physicochemical surface properties and the protein affinity and/or the abundance in serum. The determining factors with regard to the NP were: NP material, size, surface charge, surface curvature, surface ligand modifications and their stability during incubation. Concerning the proteins it was found that abundant proteins bind first but become replaced over time by proteins of higher affinity to the NP surface. When applying high sensitivity proteomics technologies such as LC–MS/MS, surprisingly many proteins were detected within the different coronas of different NP. Besides opsonizing proteins, other proteins were identified such as transport and immuno-related proteins. This plethora of corona proteins will mediate the NP navigation within body fluids and across membranes and provides a plausible interpretation of the very many sites of retention and accumulation of NP in various organs and tissues of the organism.
